# *CASC15* polymorphisms are correlated with cervical cancer susceptibility in Chinese women

**DOI:** 10.1002/mgg3.1246

**Published:** 2020-04-23

**Authors:** Ziying Gao, Zichao Xiong, Yao Sun, Jiamin Wu, Jianfeng Liu, Yuanwei Liu, Haiyue Li, Bin Li, Tianbo Jin

**Affiliations:** ^1^ Department of Clinical Laboratory Baoji Central Hospital Baoji Shaanxi China; ^2^ Key Laboratory of Resource Biology and Biotechnology in Western China Ministry of Education School of Medicine Northwest University Xi'an Shaanxi China; ^3^ Key Laboratory of Molecular Mechanism and Intervention Research for Plateau Diseases of Tibet Autonomous Region School of Medicine Xizang Minzu University Xianyang Shaanxi China; ^4^ School of Basic Medical Sciences Xizang Minzu University Xianyang Shaanxi China

**Keywords:** *CASC15*, cervical cancer, long noncoding RNA, polymorphism

## Abstract

**Background:**

Cervical cancer is a frequent, common cancer in women, and causes high cancer‐related deaths among women in our world. Accumulating studies provided an important evidence for long noncoding RNA (lncRNA) polymorphisms in the susceptibility of various cancer. Here, we recruited 494 cervical cancer cases and 504 unrelated controls to assess the relationship between *CASC15* (OMIM# 616610) polymorphisms and cervical cancer susceptibility.

**Methods:**

Agena MassARRAY platform was conducted to genotype *CASC15* polymorphisms. Odds ratios (ORs) and 95% confidence intervals (CIs) were analyzed through logistic regression to adjust for confounding factors, such as age and gender.

**Results:**

Our study suggested that rs12212674 (NC_000006.12:g.22086845T>A) “A” allele was significantly associated with an increased risk of cervical cancer (OR = 1.31, 95% CI = 1.01–1.69, *p* = .041). The result was demonstrated in the log‐additive model (OR = 1.32, 95% CI = 1.02–1.72, *p* = .037). After age stratification, we also found that the “TT” genotype of rs4712653 (NC_000006.11:g.22125964T>C) in *CASC15* was interaction with a higher cervical cancer risk in subjects aged ≤51 years in the co‐dominant model (OR = 2.08, 95% CI = 1.02–4.25, *p* = .044) and the recessive model (OR = 2.11, 95% CI = 1.05–4.24, *p* = .036). Whereas no significant correlation was found among other SNPs of *CASC15* polymorphisms and the risk of cervical cancer. MDR analysis illustrated that the interaction between rs7740084 (NC_000006.11:g.21727531G>A), rs1555529 (NC_000006.11:g.21691704A>G), and rs12212674 had a certain effect on the progress of cervical cancer.

**Conclusion:**

Our results revealed a potential interaction between *CASC15* polymorphisms and cervical cancer susceptibility. The results provided important insights into *CASC15* function in the development of cervical cancer.

## Graphical abstract

1. Cancer susceptibility candidate 15 (CASC15) is a highly active lncRNA and involved in the development of cancers.2. Our study firstly showed that there is a potential interaction between CASC15 polymorphisms and cervical cancer susceptibility.

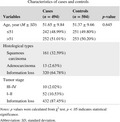

## 1 INTRODUCTION

Cervical cancer is the fourth common gynecological cancer, which causes high cancer‐related deaths among women, especially in developing countries (McGuire, 2016). It is estimated that 266,000 deaths and 528,000 new cases occurred globally each year (Ferlay et al., 2015). The increasing researches have provided evidence that the human papillomavirus (HPV) is a primary risk factor of cervical cancer. However, most women are temporarily infected with HPV, and only a small portion of them will develop cervical cancer, indicating that other factors also played a crucial role in the development of cervical cancer (Hariri et al., 2011). Plenty of epidemiological studies suggested that environment stimulation and individual heritability were associated with the occurrence and development of cervical cancer (Liu, Zhou, Su, & Zhang, 2019; Liu, Lyu, Zhang, Sheng, & Tang, 2017). Single nucleotide polymorphisms (SNPs) are considered to be correlated with various cancers, such as lung cancer (Zhong et al., 2013), breast cancer (Xia et al., 2015), gastric cancer (Yuan et al., 2012), and cervical cancer (Gao et al., 2019). Thus, in order to develop effective personalized medicine, the detection of SNPs is very important for the susceptibility of cervical cancer.

Long noncoding RNAs (lncRNAs) are members of these ncRNA families. They are a type of RNA transcripts with more than 200 nucleotides and no protein‐coding ability. Numerous studies have revealed that lncRNAs are involved in gene function via multiple mechanisms, such as transcriptional regulation, chromatin remodeling, and genetic imprinting (Glusman et al., 2006). Moreover, there are increasing studies indicated that lncRNA polymorphism was correlated with various cancer risks (Tang et al., 2017). Cancer susceptibility candidate 15 (*CASC15*, OMIM# 616610) is a highly active lncRNA, also known as LINC00340 (Yin, Zhao, Li, & Yin, 2018). Jing et al reported that the high expression of *CASC15* affected the development of gastric cancer (Yao, Tang, Zhu, & Jing, 2017). A research consisting of 118 cases and 281 healthy controls confirmed that *CASC15* polymorphisms significantly reduced the risk of neuroblastoma among Chinese children (Zhang et al., 2017). Shan et al reported that patient with a higher expression of lncRNA *CASC15* has a poor prognosis. And, *CASC15* knockdown can inhibit cell proliferation, cell cycle progression, cell invasion ability, and epithelial‐mesenchymal transition (EMT) signaling pathway. However, no study revealed that the correlation between *CASC15* polymorphisms and cervical cancer susceptibility in the Chinese Han women.

Thus, we investigated the relationship between *CASC15* polymorphisms and cervical cancer risk in the Chinese women. The results provided important insights into lncRNA *CASC15* function in the development of cervical cancer.

## 2 MATERIALS AND METHODS

### 2.1 Study subjects

A total of 494 cases, newly diagnosed and histologically validated cervical cancer patients, were recruited from Shaanxi Provincial Cancer Hospital in the current study. Patients who have history of gynecologic diseases were excluded from our research. About 504 age and gender‐matched unrelated controls were randomly recruited from the health care in the same hospital during the same time. The following are the exclusion criteria of controls: no gynecological neoplasm, no endometriosis, no other history of solid cancers, and no immune disorders. In addition, demographic and clinical factors, including age, histological types, tumor stage, carcinoembryonic antigen (CEA), serum ferritin (SF), tumor necrosis factor (TNF), sugar antigen (CA), alpha fetoprotein (AFP), and human epididymis protein (HE), were collected. The present study was approved by the clinical ethical committee of the Shaanxi Provincial Cancer Hospital. In addition, the detail researches of this study and the written informed were also signed.

### 2.2 DNA extraction and genotyping

Peripheral blood samples (5 ml) were obtained from each individual. DNA was isolated from the blood samples by GoldMag DNA purification kit (GoldMag) following the manufacturer's instructions. Subsequently, NanoDrop 2000 platform (Thermo Fisher Scientific) was performed to evaluate DNA purity and concentration. Based on the minor allele frequency (MAF) > 5% and Hardy–Weinberg equilibrium (HWE) > 0.001%, *CASC15* SNPs were selected in the global population from the 1,000 Genomes Project data (http://www.internationalgenome.org/). When *r* ^2^ (the measure value of LD) > 0.8, the SNP can represent all the polymorphisms in a block. Ultimately, six SNPs including rs1555529 (NC_000006.11:g.21691704A>G), rs7740084 (NC_000006.11:g.21727531G>A), rs1928168 (NC_000006.11:g.22017738T>C), rs12212674 (NC_000006.12:g.22086845T>A), rs4712653 (NC_000006.11:g.22125964T>C), and rs9393266 (NC_000006.11:g.22220860C>T) were finally selected in the present study. Furthermore, the Agena Bioscience Assay Design Suite V2.0 software and the MassARRAY iPLEX platform (Agena Bioscience) designed the amplification and extension primers and conducted the SNPs' genotyping. In the end, all data were performed by Agena Bioscience TYPER version 4.0 software.

### 2.3 Statistical analysis

Statistical analyses were performed by SPSS 20.0 software. The demographics and genotype frequencies were assessed by χ^2^ tests among all individuals. We applied χ^2^ test to evaluate if the candidate SNPs deviated from Hardy–Weinberg equilibrium. The relationship between *CASC15* polymorphisms and the risk of cervical cancer was assesses through odds ratio (ORs) and 95% confidence interval (CI). We also detected the potential interaction between selected SNPs and cervical cancer risk using the MDR software package. All *p*‐values were two‐tailed and *p* < .05 was statistically significant.

## 3 RESULTS

### 3.1 Characteristics of study individuals

All of 494 cervical cancer cases and 504 unrelated controls were involved in the research. The detail characteristics of the individuals are listed in Table 1. We found that the mean age of cases and controls were 51.65 ± 9.84 years and 51.37 ± 9.66 years, respectively. No difference between cervical cancer cases and healthy controls in age was observed (*p* = .645).

**TABLE 1:** Characteristics of cases and controls

Variables	Cases (*n* = 494)	Controls (*n* = 504)	*p‐*value
Age, year (*M* ± *SD*)	51.65 ± 9.84	51.37 ± 9.66	0.645
≤51	242 (48.99%)	251 (49.80%)	
>51	252 (51.01%)	253 (50.20%)	
Histological types			
Squamous carcinoma	161 (32.59%)		
Adenocarcinoma	13 (2.63%)		
Information loss	320 (64.78%)		
Tumor stage			
Ⅲ–Ⅳ	10 (2.02%)		
Ⅰ–Ⅱ	52 (10.53%)		
Information loss	432 (87.45%)		

Notes

*p* values were calculated from χ^2^ test. *p* < .05 indicates statistical significance.

Abbreviation: *SD*, standard deviation.

### 3.2 *CASC15* polymorphisms and cervical cancer risk

The detail information (such as the position, MAF, and HWE *p* value) of the candidate SNPs in participants is summarized in Table 2. The results confirmed that the genotype frequencies of the selected six SNPs were all in accord with Hardy–Weinberg equilibrium (*p* > .05).

**TABLE 2:** Basic characteristics and allele frequencies among *CASC15* SNPs

SNP	Chr	Allele	MAF		HWE *p‐*value
			Case	Control	
rs1555529	6	A/G	0.36	0.38	.849
rs7740084	6	G/A	0.44	0.45	.928
rs1928168	6	C/T	0.20	0.19	.319
rs12212674	6	A/T	0.15	0.12	.209
rs4712653	6	T/C	0.27	0.26	.489
rs9393266	6	T/C	0.48	0.50	.422

Notes

Hardy–Weinberg equilibrium; The GenBank reference of the *CASC15* was NC_000006.12. *p* values were calculated with two‐sided χ^2^.

Abbreviation: MAF, minor allele frequency; SNP, single nucleotide polymorphism.

The allele and genotype frequencies distribution were analyzed by χ^2^ tests and odds ratios (ORs) to assess the correlation with cervical cancer risk. All results are displayed in Table 3. We validated that the “A” allele of rs12212674 increased the risk of cervical cancer (OR = 1.31, 95% CI = 1.01–1.69, *p* = .041), when compared with the “T” allele. In addition, the relationship between *CASC15* polymorphisms and cervical cancer sensibility was examined in four genetic models. We observed that rs12212674 of *CASC15* improved cervical cancer risk (OR = 1.32, 95% CI = 1.02–1.72, *p* = .037) in the log‐additive model. However, no significant interaction was confirmed among the remaining SNPs of *CASC15* polymorphisms and the risk of cervical cancer.

**TABLE 3:** The association between six SNPs within the *CASC15* and the risk of cervical cancer

SNP	Model	Genotype	Cases	Controls	OR (95% CI)	*p‐*value
rs1555529	Allele	G	637	630	1	
		A	351	378	0.92 (0.77–1.10)	.360
	Co‐dominant	G/G	212	198	1	
		G/A	213	234	0.85 (0.65–1.11)	.227
		A/A	69	72	0.89 (0.61–1.31)	.564
	Dominant	G/G	212	198	1	
		G/A‐A/A	282	306	0.86 (0.67–1.11)	.236
	Recessive	G/G‐G/A	425	432	1	
		A/A	69	72	0.97 (0.68–1.39)	.885
	Log‐additive	—	—	—	0.92 (0.77–1.10)	.361
rs7740084	Allele	A	556	549	1	
		G	448	439	0.99 (0.83–1.18)	.933
	Co‐dominant	A/A	158	153	1	
		A/G	233	250	0.90 (0.68–1.20)	.483
		G/G	103	99	1.01 (0.71–1.43)	.976
	Dominant	A/A	158	153	1	
		G/A‐G/G	336	349	0.93 (0.71–1.22)	.607
	Recessive	A/A‐G/A	391	403	1	
		G/G	103	99	1.07 (0.79–1.46)	.669
	Log‐additive	—	—	—	0.99 (0.83–1.18)	.925
rs1928168	Allele	T	794	810	1	
		C	194	196	1.01 (0.81–1.26)	.932
	Co‐dominant	T/T	321	322	1	
		T/C	152	166	0.92 (0.70–1.20)	.532
		C/C	21	15	1.40 (0.71–2.76)	.336
	Dominant	T/T	321	322	1	
		T/C‐C/C	173	181	0.96 (0.74–1.24)	.742
	Recessive	T/T‐T/C	473	488	1	
		C/C	21	15	1.44 (0.73–2.82)	.292
	Log‐additive	—	—	—	1.01 (0.81–1.26)	.943
rs12212674	Allele	T	887	835	1	
		A	121	149	**1.31 (1.01–1.69)**	**.041**
	Co‐dominant	T/T	353	387	1	
		T/A	129	113	1.25 (0.94–1.68)	.128
		A/A	10	4	2.74 (0.85–8.81)	.091
	Dominant	T/T	353	387	1	
		T/A‐A/A	139	117	1.31 (0.98–1.74)	.068
	Recessive	T/T‐T/A	482	500	1	
		A/A	10	4	2.59 (0.81–8.32)	.110
	Log‐additive	—	—	—	**1.32 (1.02–1.72)**	**.037**
rs4712653	Allele	C	718	741	1	
		T	270	263	1.06 (0.87–1.29)	.568
	Co‐dominant	C/C	260	270	1	
		C/T	198	201	1.02 (0.79–1.33)	.865
		T/T	36	31	1.21 (0.72–2.01)	.472
	Dominant	C/C	260	270	1	
		C/T‐T/T	234	232	1.05 (0.82–1.34)	.717
	Recessive	C/C‐C/T	458	471	1	
		T/T	36	31	1.19 (0.73–1.96)	.485
	Log‐additive	—	—	—	1.06 (0.87–1.30)	.565
rs9393266	Allele	C	506	504	1	
		T	496	472	0.96 (0.80–1.14)	.612
	Co‐dominant	C/C	122	123	1	
		C/T	260	260	1.01 (0.75–1.37)	.943
		T/T	106	118	0.91 (0.63–1.30)	.599
	Dominant	C/C	122	123	1	
		C/T‐T/T	366	378	0.98 (0.73–1.31)	.884
	Recessive	C/C‐C/T	382	383	1	
		T/T	106	118	0.90 (0.67–1.21)	.490
	Log‐additive	—	—	—	0.95 (0.80–1.14)	.609

Notes

The GenBank reference of the *CASC15* was NC_000006.12. Bold type indicates statistical significance (*p* < .05).

Abbreviations: CI, confidence interval; OR, odds ratio; SNP, single nucleotide polymorphism.

### 3.3 Stratified analysis of *CASC15* polymorphisms and cervical cancer risk

To control potential confounders, the participants were stratified by age. We demonstrated that rs4712653 “TT” genotype was associated with an increased risk of cervical cancer in aged ≤51 population (co‐dominant: OR = 2.08, 95% CI = 1.02–4.25, *p* = .044; recessive: OR = 2.11, 95% CI = 1.05–4.24, *p* = .036, respectively). In terms of subjects with age >51 years, there was no statistically significant association between the candidate SNPs of *CASC15* polymorphisms and cervical cancer risk (*p* > .05). The results are shown in Table 4.

**TABLE 4:** The association between the *CASC15* polymorphisms and cervical cancer risk stratified by age

SNP	Model	Genotype	≤51		>51	
			OR (95%CI)	*p‐*value	OR (95%CI)	*p‐*value
rs1555529	Allele	G	1			
		A	0.96 (0.74–1.25)	.760	0.88 (0.68–1.13)	.321
	Co‐dominant	G/G	1			
		G/A	0.79 (0.54–1.16)	.223	0.76 (0.44–1.30)	.313
		A/A	1.06 (0.62–1.82)	.835	0.91 (0.62–1.33)	.617
	Dominant	G/G	1			
		G/A‐A/A	0.85 (0.59–1.21)	.369	0.87 (0.61–1.25)	.447
	Recessive	G/G‐G/A	1			
		A/A	1.19 (0.72–1.98)	.496	0.80 (0.48–1.32)	.375
	Log‐additive	—	0.96 (0.75–1.24)	.767	0.88 (0.68–1.13)	.321
rs7740084	Allele	A	1			
		G	0.97 (0.75–1.24)	.792	1.02 (0.79–1.30)	.899
	Co‐dominant	A/A	1			
		A/G	1.05 (0.70–1.57)	.818	1.08 (0.66–1.79)	.754
		G/G	0.92 (0.55–1.52)	.733	0.78 (0.52–1.17)	.230
	Dominant	A/A	1			
		G/A–G/G	1.01 (0.69–1.47)	.970	0.86 (0.59–1.26)	.439
	Recessive	A/A‐G/A	1			
		G/G	0.89 (0.57–1.39)	.612	1.27 (0.82–1.95)	.281
	Log‐additive	—	0.97 (0.76–1.24)	.797	1.02 (0.79–1.30)	.901
rs1928168	Allele	T	1			
		C	0.93 (0.68–1.28)	.657	1.09 (0.80–1.49)	.580
	Co‐dominant	T/T	1			
		T/C	0.89 (0.61–1.31)	.560	1.84 (0.72–4.74)	.206
		C/C	1.00 (0.37–2.73)	1.000	0.94 (0.64–1.38)	.754
	Dominant	T/T	1			
		T/C–C/C	0.90 (0.62–1.31)	.585	1.01 (0.70–1.46)	.951
	Recessive	T/T–T/C	1			
		C/C	1.04 (0.38–2.81)	.941	1.88 (0.74–4.80)	.187
	Log‐additive	—	0.93 (0.67–1.28)	.651	1.09 (0.80–1.48)	.606
rs12212674	Allele	T	1			
		A	1.19 (0.83–1.71)	.335	1.45 (1.00–2.10)	.051
	Co‐dominant	T/T	1			
		T/A	1.08 (0.72–1.61)	.722	1.85 (0.44–7.85)	.405
		A/A	5.4 (0.62–46.76)	.126	1.47 (0.97–2.25)	.072
	Dominant	T/T	1			
		T/A– A/A	1.14 (0.77–1.70)	.513	1.50 (0.99–2.26)	.055
	Recessive	T/T – T/A	1			
		A/A	5.29 (0.61–45.71)	.130	1.69 (0.40–7.15)	.477
	Log‐additive	—	1.21 (0.83–1.75)	.319	1.45 (0.99–2.11)	.054
rs4712653	Allele	C	1			
		T	1.21 (0.91–1.60)	.188	0.93 (0.70–1.23)	.618
	Co‐dominant	C/C	1			
		C/T	0.97 (0.67–1.41)	.867	0.61 (0.28–1.34)	.218
		T/T	**2.08 (1.02–4.25)**	**.044**	1.08 (0.75–1.55)	.697
	Dominant	C/C	1			
		C/T‐T/T	1.10 (0.77–1.57)	.608	1.00 (0.71–1.43)	.980
	Recessive	C/C‐C/T	1			
		T/T	**2.11 (1.05–4.24)**	**.036**	0.59 (0.27–1.28)	.181
	Log‐additive	—	1.20 (0.91–1.59)	.195	0.93 (0.69–1.24)	.616
rs9393266	Allele	C	1			
		T	1.08 (0.84–1.38)	.574	1.17 (0.92–1.51)	.204
	Co‐dominant	C/C	1			
		C/T	1.37 (0.89–2.12)	.157	1.38 (0.83–2.28)	.212
		T/T	1.16 (0.68–1.96)	.585	1.04 (0.67–1.61)	.861
	Dominant	C/C	1			
		C/T‐T/T	1.31 (0.86–1.98)	.210	1.14 (0.75–1.73)	.532
	Recessive	C/C‐C/T	1			
		T/T	0.93 (0.61–1.43)	.741	1.34 (0.90–2.01)	.154
	Log‐additive	—	1.08 (0.83–1.41)	.555	1.18 (0.91–1.52)	.205

Note

The GenBank reference of the *CASC15* was NC_000006.12. Bold type indicates statistical significance (*p* < .05).

Abbreviations: CI, confidence interval; OR, odds ratio; SNP, single nucleotide polymorphism.

#### 3.3.1 SNP–SNP interaction and cervical cancer risk

MDR analysis is summarized in Table 5. The interaction information analysis showed that the interaction between rs7740084, rs1555529, and rs12212674 had a certain effect on the progress of cervical cancer. The combination of high‐risk and low‐risk genotypes was determined according to the threshold, and we can see that the TT genotype of rs12212674, AA genotype of rs1555529, and AA genotype of rs7740084 had a higher risk of cervical cancer. And, the testing accuracy and cross‐validation consistency of the two locus model (rs7740084 and rs1555529) and the three locus model (rs7740084, rs1555529, and rs12212674) was 54.39%, 4/10 and 56.70%, 4/10, respectively, which was significant.

**TABLE 5:** Predication of cervical cancer risk factors in MDR analysis

Best model	Training accuracy (%)	Testing accuracy (%)	Cross‐validation consistency	χ^2^	*p*
rs12212674	52.72	49.03	7/10	3.695	.055
rs1555529|rs7740084	54.39	44.66	4/10	6.344	**.012**
rs7740084|rs1555529|rs12212674	56.70	46.95	4/10	16.447	**<.000**

Notes

*p*‐values were calculated by Pearson χ^2^ test. Bold type indicates statistical significance (*p* < .05).

Abbreviation: MDR, multifactor dimensionality reduction.

## 4 DISCUSSION

Cervical cancer is a common frequent cancer among women. It was reported that cervical cancer has a high mortality worldwide (McGuire, 2016). Recent evidences indicated that genetic factors played a crucial role in the development of cervical cancer (Liu et al., 2019). The case‐control study indicated that rs12212674 of *CASC15* polymorphisms was significantly associated with an increased risk of cervical cancer (*p* = .041). The “TT” genotype of rs4712653 was related to improved cervical cancer risk in aged ≤51 populations (*p* = .036).

Growing researches have confirmed that lncRNAs may have an effect on various malignant tumors, including cervical cancer, through regulating cell differentiation, proliferation, apoptosis, migration, and invasion (Camacho, Choudhari, & Gadad, 2018; Tang et al., 2017). Gao et al suggested that lncRNA *PANDAR* (OMIM# 617179) was significantly associated with cervical cancer, and upregulation of lncRNA *PANDAR* can promote cervical cancer cell proliferation (Huang, Xie, Ma, Zhao, & Gao, 2017). Recent study has shown that lncRNA *HOTAIR* (OMIM# 611400) was heightened in cervical cancer tissues and correlated with poor prognosis of cervical cancer patients (Li, Wang, Yu, Dong, & Qiu, 2015). In addition, a study including 1,209 patients and 1,348 healthy controls also demonstrated that rs7958904 of *HOTAIR* might affect cervical cancer susceptibility by modulation of cervical cancer cell proliferation (Jin et al., 2017). Wang et al suggested that rs11655237 (NC_000017.10:g.70400166C>T) of LINC00673 (OMIM# 617079) polymorphism was correlated with cervical cancer risk among Chinese population (Wang & Luo, 2018). Moreover, numerous lncRNAs are abnormally expressed in cervical cancer (Peng, Yuan, Jiang, Tang, & Li, 2016). To date, no studies have reported the correlation between lncRNA *CASC15* polymorphisms and cervical cancer susceptibility in Chinese females.

Several studies have indicated that *CASC15* expression was associated with different human diseases, including neuroblastoma (Russell et al., 2015), hepatocellular carcinoma (He, Zhang, et al., 2017), acute leukemia (Fernando et al., 2017), and gastric cancer (Yao et al., 2017; Yuan et al., 2012). Two genome‐wide association studies (GWASs) have verified that *CASC15* rs4712653 T>C was correlated with a decreased risk of neuroblastoma in Chinese children (He et al., 2016; He, Zou, et al., 2017). To our best knowledge, we firstly demonstrated that *CASC15* polymorphisms were interaction with cervical cancer susceptibility in Chinese women. We observed that *CASC15* rs4712653 “TT” genotype was associated with the risk of cervical cancer in aged ≤51 people (*p* = .036).

In summary, the results revealed the role of *CASC15* polymorphisms in cervical cancer sensibility among Chinese women. Besides, detail molecular mechanism studies to further explore the role of *CASC15* variants in increasing cervical cancer risk were necessary to be performed.

## CONFLICT OF INTEREST

The authors have declared that they have no conflict of interest.

## AUTHOR CONTRIBUTIONS

ZYG completed genotyping and performed the manuscript. ZCX and YS took part in genotyping. JM W, JF L, YW L, and HYL participated in the statistical analysis of the data and modified the manuscript. BL and TBJ designed the study, co‐supervised the work, and finalized the manuscript. All the authors have read and approved the final manuscript.

## Data Availability

Not applicable.
